# Genome sequencing of an ESBL-producing *E. coli* BLRI_001 strain harboring *bla*TEM-176 and *bla*EC-15 variants isolated from a live bird market in Dhaka, Bangladesh

**DOI:** 10.1128/mra.00839-25

**Published:** 2025-09-12

**Authors:** Md Hafizur Rahman, Md Zulfekar Ali, Kazi Injamamul Hoque, Mahmudul Hasan

**Affiliations:** 1 Animal Health Research Division, Bangladesh Livestock Research Institute 560480, Savar, Dhaka, Bangladesh; 2 AMR Reference Laboratory (Research), Bangladesh Livestock Research Institute 560480, Savar, Dhaka, Bangladesh; 3 Food Safety and One Health Laboratory, International Centre for Diarrhoeal Disease Research 56291, Dhaka, Bangladesh; University of Maryland School of Medicine, Baltimore, Maryland, USA

**Keywords:** *Escherichia coli*, extended-spectrum beta lactamase (ESBL), live bird market

## Abstract

We report the genome sequence of an ESBL-producing *Escherichia coli* BLRI_001 strain (O8:H11, ST6018, phylogroup A) isolated from a live bird market in Dhaka, Bangladesh, harboring *bla*TEM-176, *bla*EC-15, with 6 replicon plasmids, 13 virulence genes, 11 antibiotic resistance genes, and 2 biocide resistance genes.

## ANNOUNCEMENT

Antibiotic-resistant *Escherichia coli* strains pose a significant global health threat, particularly in animal and public health settings. In 2024, *E. coli* BLRI_001 strain was isolated from a live bird market (poultry feces) in Dhaka by culture on Eosin methylene Blue (EMB) agar (Oxoid, UK), followed by incubation at 37°C for 24 h. The colonies, showing metallic green sheens on the plate, underwent VITEK 2 system and polymerase chain reaction (PCR) to confirm the *E. coli* (1). The *E. coli* BLRI_001 strain was cultured in nutrient broth (Oxoid, UK) aerobically overnight at 37°C, and the DNA extraction from the cultured broth was done using the DNeasy Blood and Tissue Kit (Qiagen, Hilden, Germany). Subsequently, the DNA library was prepared with the Nextera DNA Flex Library Prep Kit (Illumina, San Diego, CA, USA). Genome sequencing was conducted using the Illumina NextSeq2000 platform, generating paired-end reads with a 2 × 150 bp length and 18,601,764 reads. The genome assembly was performed by Unicycler v0.4.8 (2), followed by the trimming of raw paired-end reads (*n* = 9,300,882) using Trimmomatic v0.39 (3) to remove Illumina adapters, known Illumina artifacts, and phiX reads from the data set. Quality assessment was performed through FastQC v0.11.7 (4). Genome annotation was performed using the NCBI Prokaryotic Genome Annotation Pipeline (PGAP v6.6) (5). Sequence type was predicted using MLST v.2.0 (6) using the Center for Genomic Epidemiology database (https://genomicepidemiology.org/services/), serotype by SerotypeFinder v.2.0 (7) and SeroPred tool (8), the pathogenicity index by PathogenFinder v.1.1 (9), CRISPR arrays and prophages by CRISPRimmunity (10), plasmids by PlasmidFinder v.2.1 (11) and MobSuite 3.1.9 mob-typer (12), antibiotic resistance genes (ARGs) by the AMRFinderPlus 3.11.20 (12), ResFinder (v.4.6.0) (13, 14) and CARD (v.6.0.3) (15), virulence factor genes (VFGs) by the virulence factor database with VFanalyzer VFDB 2022 (16), and metabolic functional features were identified by RAST (v.2.0) (17). Unless otherwise noted, default parameters were used for all software.

The total length of the *E. coli* BLRI_001 strain genome was 4,729,543 bp with 133 contigs. In RAST, the assembled genome showed 377 subsystems with 32% coverage and 1,556 genes. The data generated from RAST were submitted in the open access platform-ZENODO (https://doi.org/10.5281/zenodo.16812166). The general characteristics are indicated in Table 1.

**TABLE 1:** Summary of general characteristics of *E. coli* BLRI_001 strain

Attributes	Values	
Total genes	4,618	
Coding genes	4,371	
Contig N50	70,490 bp	
G + C content	50.73%	
Serotype	O8:H11	
Genome coverage	36.0×	
RNA genes	52	
tRNA genes	45	
rRNA	2	
Non-coding RNA	6	
CRISPR arrays	2	
Replicon plasmids	Replicon types	Contig number
	IncFIB	71
	IncHI1B	104
	IncX1	120
	IncX2	118
	IncN	132
	ColRNAI rep cluster 1987	20
Predicted antibiotic resistance genes (ARGs) by ResFinder	7	
Predicted ARGs by AMRFinderPlus	11	
Predicted ARGs by CARD	58	
Predicted virulence genes	13	
Pathogenicity index	0.943	
Sequence type	ST6018	
SRA	SRX29634662	
BioSample accession no.	SAMN46911086	
BioProject accession no.	PRJNA1225933	
GenBank accession no.	JBLUNT000000000	
